# Efficient path-based computations on pedigree graphs with compact encodings

**DOI:** 10.1186/1471-2105-13-S3-S14

**Published:** 2012-03-21

**Authors:** Lei Yang, En Cheng, Z Meral Özsoyoğlu

**Affiliations:** 1 Electrical Engineering and Computer Science Department, Case Western Reserve University, Cleveland, OH, USA

## Abstract

A pedigree is a diagram of family relationships, and it is often used to determine the mode of inheritance (dominant, recessive, etc.) of genetic diseases. Along with rapidly growing knowledge of genetics and accumulation of genealogy information, pedigree data is becoming increasingly important. In large pedigree graphs, path-based methods for efficiently computing genealogical measurements, such as inbreeding and kinship coefficients of individuals, depend on efficient identification and processing of paths. In this paper, we propose a new compact path encoding scheme on large pedigrees, accompanied by an efficient algorithm for identifying paths. We demonstrate the utilization of our proposed method by applying it to the inbreeding coefficient computation. We present time and space complexity analysis, and also manifest the efficiency of our method for evaluating inbreeding coefficients as compared to previous methods by experimental results using pedigree graphs with real and synthetic data. Both theoretical and experimental results demonstrate that our method is more scalable and efficient than previous methods in terms of time and space requirements.

## Introduction

With the rapidly expanding field of medical genetics and genetic counselling, genealogy information is becoming increasingly abundant. In January 2009, the U.S. Department of Health and Human Services released an updated and improved version of the Surgeon General's Web-based family health history tool [1]. This Web-based tool makes it easy for users to record their family health history. Large extended human pedigrees are very informative for linkage analysis. Pedigrees including thousands of members in 10-20 generations are available from genetically isolated populations [2,3].

The Utah Population Database [4] with 1.6 million genealogy records is one of the large, heavily used pedigree data collections. The Jagelman Registries of Cleveland Clinic [5] is an example of a pedigree data collection which is heavily used by medical and genetic researchers for the analysis of hereditary structure and identifying risk factors for inherited colon cancer.

Pedigrees are hierarchical structures showing hereditary relationships between individuals, and are typically represented as directed acyclic graphs (DAGs). In a pedigree graph, the set of paths to an individual from its ancestors is important for computing quantitative measures of the genetic relationship between individuals, such as inbreeding coefficients, kinship coefficients, and identity coefficients. For example, Wright's path-counting formula [6], for computing inbreeding coefficients requires all paths from a common ancestor to a given individual's parents. More specifically, the inbreeding coefficient of an individual is a function of the number and location of the common ancestors of both parents of this individual in the given pedigree. Efficient computation of inbreeding and kinship coefficients is important for large pedigrees, especially for real time applications such as genetic counselling. The inbreeding coefficient of an individual is one of the parameters in calculating the cancer risk of an individual in hereditary cancers [7]. Inbreeding is also important for wildlife conservationists and purebred livestock. Inbreeding coefficients are calculated routinely for animals included in national genetic evaluations for yield traits [8], and animal selection [8]. With the rapidly accumulation of genealogy information, there is need for scalable computation schemes due to both the increasing volume of available pedigree data, and the increasing usage of pedigree data analysis in medical genetics for hereditary diseases.

In the conference paper [9], we propose a compact encoding for pedigree graphs to be used for scalable and efficient computation of inbreeding coefficients and other genetic measures utilizing path-counting formulas on large pedigrees. The Compact Path Encoding for a directed acyclic graph (CPE) includes two parts: the first is called the Prefix-based Encoding for a Tree (PET), and the second part is the encoding for Non-Tree Edges (NTE). CPE encoding and using it in computing inbreeding coefficients has been first proposed in the conference paper [9]. In this paper, we proposed an improved algorithm which computes the inbreeding coefficient using CPE more efficiently. We provide time and space complexity analysis for CPE encoding algorithm and show that the size of the CPE code produced by the encoding algorithm is optimal. We also present experimental results evaluating the performance of our method for calculating inbreeding coefficients with all the previous methods. The main contributions of this paper are as follows:

I) A compact path encoding scheme for pedigree graphs.

II) An efficient algorithm for encoding pedigree graphs which produces compact path encoding with minimal size.

III) An efficient and scalable algorithm for calculating inbreeding coefficients on large pedigrees using the compact path encoding.

IV) Time and space complexity analysis of the inbreeding coefficient computation algorithm.

V) Experimental results demonstrating significant performance gains for calculating inbreeding coefficients as compared to other label-based schemes and traditional recursive algorithms.

While we focus only on pedigree graphs in this paper, Compact Path Encoding Scheme and the algorithm for constructing paths using node labels are also applicable to other directed acyclic graphs for efficient path manipulation.

## Problem statement

Pedigree data is represented by a directed acyclic graph, where the nodes represent individuals, and directed edges represent parent-child relationships. Given a pedigree graph, we can calculate many quantities of interest such as checking if an individual has inbreeding, computing an individual's inbreeding coefficients or computing kinship coefficients between individuals. Given an individual *n *having father *f *and mother *m*, the kinship coefficient between *f *and *m *is the probability that a gene selected randomly from *f *and a gene selected randomly from the same autosomal locus of *m *is identical by descent. Actually, the inbreeding coefficient of *n *is equal to the kinship coefficient between *f *and *m*. Alternatively, inbreeding coefficient of an individual can also be stated as the probability that both copies of any given gene of the individual are received from the same ancestor. The inbreeding coefficient can be computed using a recursive formula for computing kinship coefficients [10], or using Wright's path counting formula [6]. Given an individual *n*, Wright's formula for inbreeding coefficient *F_n _*is:

(1) $$F_{n}=\left\{\begin{array}{cc} \underset{A}{\sum }\underset{P}{\sum }\left[{\left(\frac{1}{2}\right)}^{r+s+1}\left(1+F_{A}\right)\right] & \text{if}\;n\;\text{has}\;\text{in}\;\text{breeding}\\ 0 & \text{otherwise} \end{array}\right\}$$

where *A *is the common ancestor of the parents of *n*, and *F_A _*is the inbreeding coefficient for *A*, *P *is a pair of paths that is not overlapping from the common ancestor *A *to the parents of *n*, r is the number of generations between *n *and *A *from the maternal side, and s is from the paternal side. The summation is performed over all common ancestors of the parents of *n *and for all non-overlapping pairs of paths from the common ancestor to the parents.

For applications requiring pedigree queries embedded into database queries, and pedigree data stored in a database, recursive methods for computing inbreeding coefficients tend not to be very scalable due to many database accesses. Path counting formulas on the other hand require efficiently identifying and manipulating paths.

**Figure 1:**
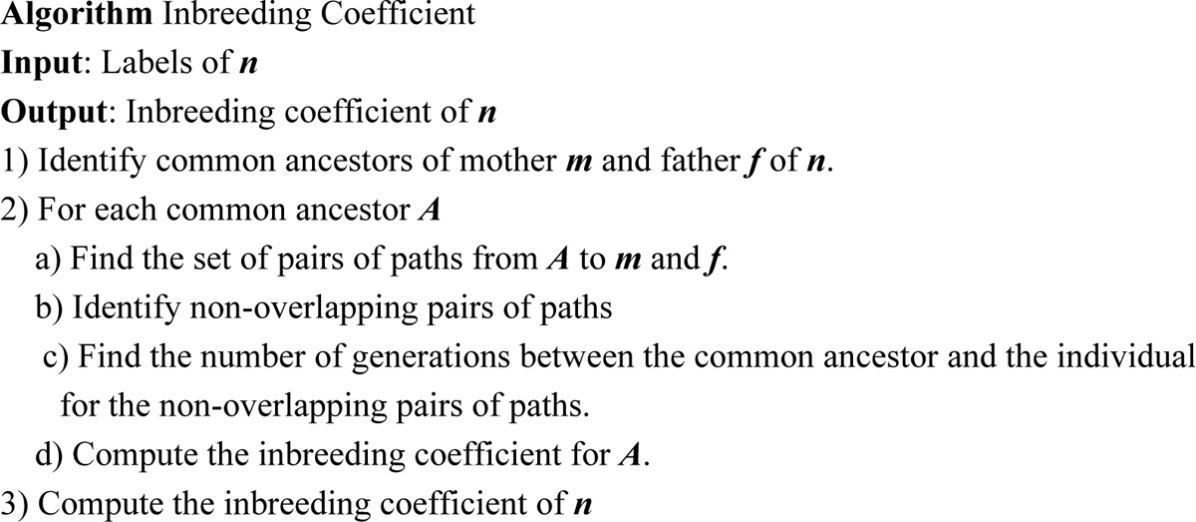
Algorithm: Inbreeding Coefficient.

In this paper, we focus on a scalable scheme for encoding pedigree graphs and efficient algorithms using path counting formulas for computations on pedigree graphs. Pedigree computations where path counting formulas can be used efficiently include inbreeding coefficients, kinship and generalized kinship coefficients [9,11], and identity coefficients which can be expressed as a linear combination of generalized kinship coefficients. Here, we use the computation of inbreeding coefficients as an example to demonstrate the efficiency of graph encoding scheme for path-based computations. The outline for inbreeding coefficients calculation using graph encoding (labelling) techniques by identifying paths is summarized in Figure 1, where the paths to an individual *n *can be identified using the labels of the node in the pedigree graph corresponding to the individual.

### Graph encoding/labelling schemes

There are three families of labelling schemes [12], namely bit-vector, prefix and interval. While very useful for answering queries requiring descendants, ancestors, siblings, etc., bit-vector [13] and interval-based schemes [14,15] are not useful for identifying path information. Since in this paper we need to identify paths, we focus only on prefix-based schemes.

Prefix-based schemes directly encode the parent of a node in a tree, as a prefix of its label using for instance a depth-first tree traversal. Dewey labelling [16] is a prefix based labelling for trees. EGDL [17] is an extension of the Dewey labelling for DAGs. NodeCodes [18,19] is a prefix based labelling scheme for directed graphs, also used to encode pedigree data and path based pedigree computations. Since NodeCodes labelling is closely related to the scheme used in this paper, next we discuss it in more detail.

### NodeCodes: a prefix based encoding

NodeCodes of a node is a set of labels each representing a path to the node from its ancestors. Given a pedigree graph, let *r *be the progenitor (i.e., the node with 0 in- degree). For each node *u *in the graph, the set of NodeCodes of *u*, denoted *NC*(*u*), are assigned using a breadth-first-search traversal starting from the source node *r *as follows:

1) If *u *is *r *then *NC*(*r*) contains only one element, the empty string.

2) Otherwise, let *u *be a node with *NC*(*u*), and *v_0_, v_1_, ... v_k _*be *u*'s children in sibling order, then for each *x *in *NC*(*u*), a code x_i_* is added to *NC*(*v_i_*), where 0 ≤ *i *≤ *k*, and * indicates the gender of the individual represented by node *v_i_*.

Basically, each node is assigned labels which are sequences of integers and delimiters, the integers represent the sibling order, and the delimiters denote the gender of the node, as well as being used to determine the generations. We use '.', ',', and ';' to denote female, male or unknown respectively.

**Figure 2:**
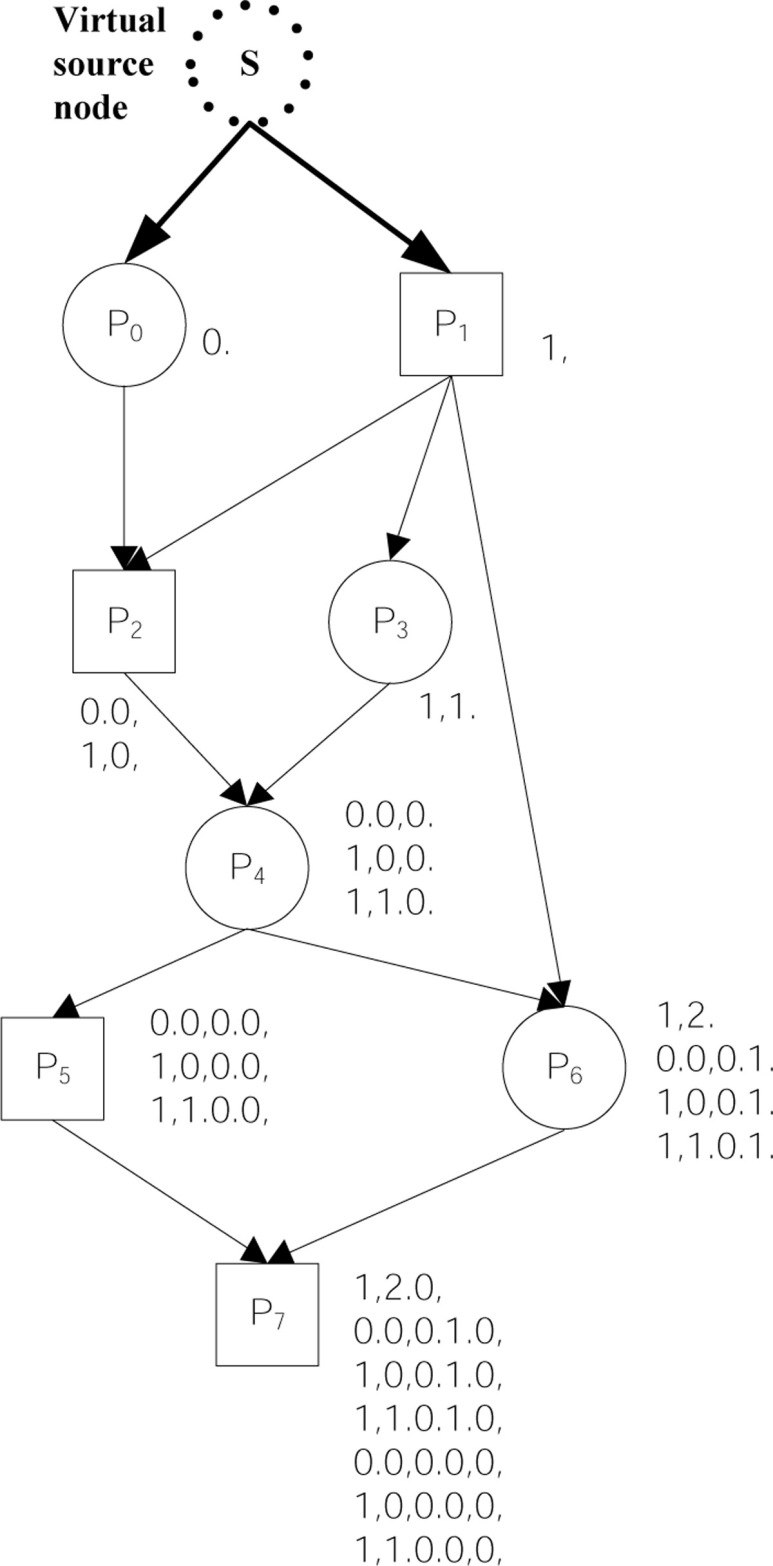
A pedigree graph with NodeCodes.

**Example 1: **Consider the pedigree graph with NodeCodes as shown in Figure 2. There are 3 and 4 paths from the root s to individuals P_5 _and P_6 _respectively. Elements of *NC*(P_5_) = {<0.0,0.0,>, <1,0,0.0,>, <1,1.0.0,>} each represents one of the 3 paths from s to P_5_. The code <1,0,0.0,> in *NC*(P_5_) represents the path <s, P_1_, P_2_, P_4_, P_5_>.

The basic steps for calculating the inbreeding coefficient of an individual *n *using NodeCodes include: 1) Find the NodeCodes of mother *m *and father *f *of individual *n*; 2) Identify common ancestors of *m *and *f*; 3) For each common ancestor *A*, identify non-overlapping pairs of paths from *A *to *m *and *f*, and compute the contribution to the inbreeding coefficients.

Given an individual *n*, with mother *m *and father *f*, identifying common ancestors of *m *and *f *requires matching *NC*(*m*) with *NC*(*f*) having the longest common prefix for matching sets. Then, the longest common prefix is used to identify the non-overlapping pairs of paths; please see our earlier work [1,18] for details.

Having one code for each path from an ancestor to the node is an advantage for NodeCodes in manipulating paths. For large pedigree graphs however, this is also the shortcoming of NodeCodes since the number of paths may increase significantly as pedigrees may have (undirected) cycles, and the number of NodeCodes for each node also increase, resulting the method to lose its scalability.

Compact Encoding described below is similar to EGDL [17], and uses one code for each node representing all paths to the node from its ancestors. The paths are generated from this code as needed, and hence resulting in significantly more compact representation of path information as compared to NodeCodes.

## Compact path encoding

In compact encoding, each node has *one *code representing all paths to the node from its ancestors, instead of having one code for each of such paths as in NodeCodes. All relevant path information can then be effectively generated using only the information in the compact encoding of the node.

Compact Path Encoding includes two parts: the first is the representation of the node in a prefix based labelling on a spanning tree of the graph where the unique progenitor, *r*, is the root. This part is the **P**refix-based **E**ncoding for **T**ree edges (or *PET*). Since there is a unique path from *r *to each node considering only the edges of the spanning tree, each node in the graph has a unique *PET *code. The second part is the encoding for **N**on-**T**ree **E**dges (or *NTE*) representing the list of non-tree edges in the paths from the root to the node. Since each node is uniquely represented by its *PET *code, each non-tree edge (*u*, *v*) is represented as (*PET*(*u*), *PET*(*v*)).

**Figure 3:**
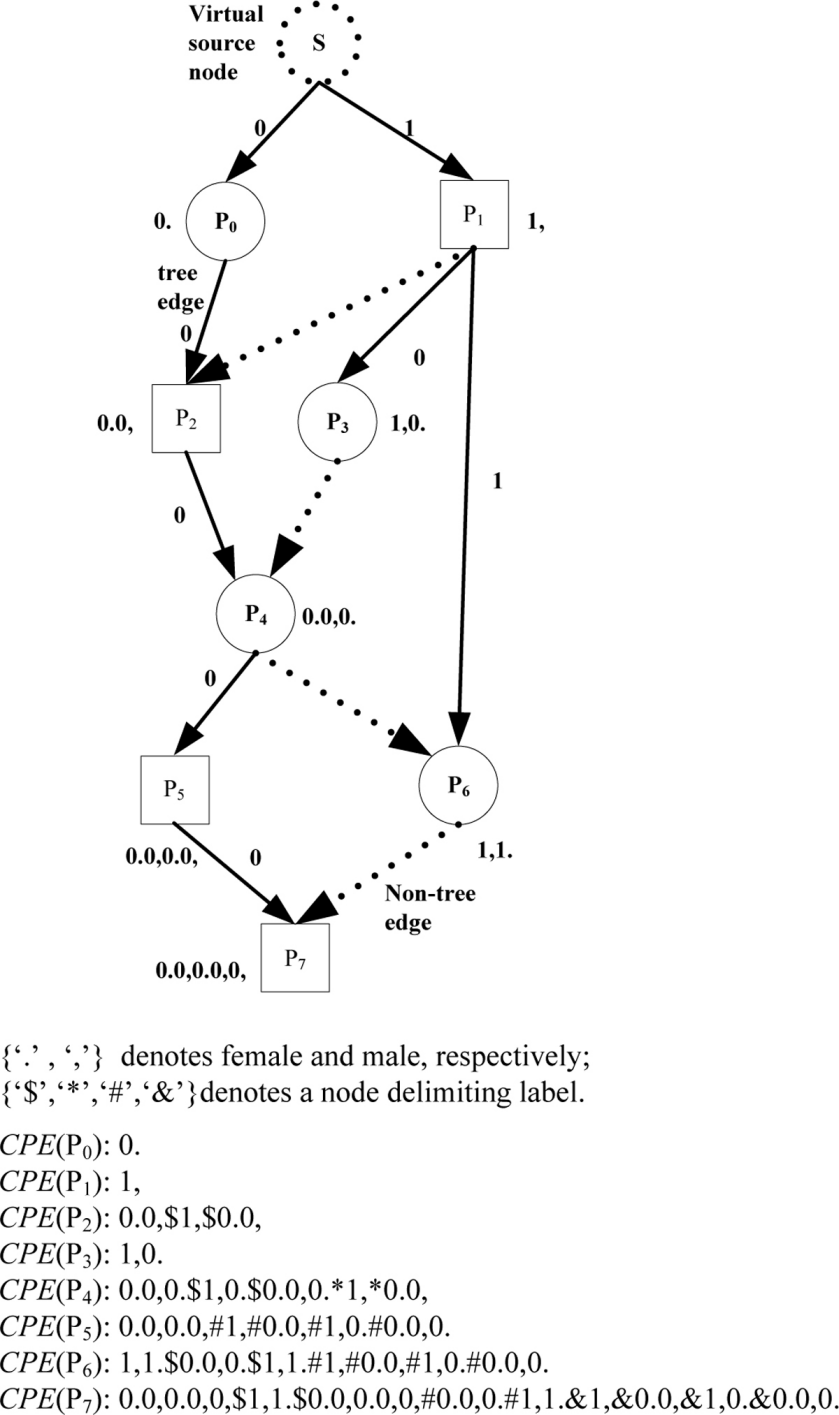
A pedigree graph with Compact Path Encoding.

**Example 2: **Consider the pedigree graph in Figure 3. Compact Encoding of node P_5_, *CPE*(P_5_) = <0.0,0.0,#1, #0.0,#1,0.#0.0,0.>, where 0.0,0.0, is *PET*(P_5_) which is the NodeCodes of P_5 _w.r.t. the spanning tree. (<1,>, <0.0,>) and (<1,0.>, <0.0,0.>) are the non-tree edges, (P_1_, P_2_) and (P_3_, P_4_) in the paths from s to P_5_, where each edge (*u*, *v*) is represented as (*PET*(*u*), *PET*(*v*)). In the *CPE *code special symbols are used as delimiters.

In the next section, Compact Path Encoding is explained with more details on the special delimiters that are used for path construction from the codes.

### Compact path encoding for DAGs

Given a single-root DAG *G*, the first step is to find a Breadth-First tree *T *from *G*. As a result, all edges in *G *are divided into tree edges and non-tree edges. After the Breadth-First tree *T *is found, the prefix-based encoding for a tree is performed on *T*. Then, for each node *v *in *G*, we obtain the path-induced subgraph of *v *in *G*.

**Definition (path-induced subgraph): **The **path-induced subgraph **of *v *in *G*, denoted as *PS*(*v*), is the unique *minimal *sub-digraph of *G *that contains all the paths in *G *from the root *r *to node *v*.

For a pedigree graph, any non-founder node is defined as having (at most two) parent nodes. Given a non-founder node *v *with parents *f *and *m*, we use a recursive formula to obtain *PS*(*v*) according to *PS*(*f*) and *PS*(*m*).

$$\begin{array}{c} PS\left(v\right)=PS\left(f\right)\cup^{G}PS\left(m\right)\cup^{G}G_{fmv}\\ G_{fmv}=\left(V=\left\{f,m,v\right\},E=\left\{\left(f,v\right),\left(m,v\right)\right\}\right) \end{array}$$

where ∪^G ^denotes a graph union operator, which operates on both the sets of *vertices *and the sets of edges of two graphs.

To generate the *CPE *label of the node *v *in *G*, denoted as *CPE*(*v*), we need to obtain all non-tree edges of *PS*(*v*), denoted by *NTE*(*v*). We use the non-tree edges of *NTE*(*f*) and *NTE*(*m*) to obtain *NTE*(*v*). More specifically, *NTE*(*v*) = *NTE*(*f*)∪*NTE*(*m*)∪{*e*} where e = (*p*,*v*) denotes the non-tree edge from one parent p (either *f *or *m*) to v.

For an edge e in NTE(v), there are four cases:

$$\begin{array}{c} case0:e=\left(p,v\right)\\ case1:e\in NTE\left(f\right)\;\text{and}\;e\notin NTE\left(m\right)\\ case2:e\in NTE\left(m\right)\;\text{and}\;e\notin NTE\left(f\right)\\ case3:e\in NTE\left(f\right)\;\text{and}\;e\in NTE\left(m\right) \end{array}$$

Any edge *e *= (*u*, *v*) in *G *can be represented by a pair of *PET*(*u*) and *PET*(*v*). More specifically, the *CPE *encoding for a non-tree edge *e *∈ *NTE*(*v*) is represented as

$$edgeCPE\left(e\right)=\left\{\begin{array}{c} {}^{\prime }{\$}^{\prime }+PET\left(v_{1}\right){+}^{\prime }{\$}^{\prime }+PET\left(v_{2}\right)\;\text{if}\;case0\\ {}^{\prime }{*}^{\prime }+PET\left(v_{1}\right){+}^{\prime }{*}^{\prime }+PET\left(v_{2}\right)\;\text{if}\;case1\\ {}^{\prime }{\#}^{\prime }+PET\left(v_{1}\right){+}^{\prime }{\#}^{\prime }+PET\left(v_{2}\right)\;\text{if}\;case2\\ {}^{\prime }{\&}^{\prime }+PET\left(v_{1}\right){+}^{\prime }{\&}^{\prime }+PET\left(v_{2}\right)\text{if}\;case3 \end{array}\right)$$

where {'$', '*', '#', '&'} denote *PET *delimiters.

Finally, for a node *v *which has the non-tree edges {*e_1_*, *e_2 _*... *eg*} in *PS*(*v*), the *CPE *encoding for *v *is represented as

$$CPE\left(v\right)=PET\left(v\right)+edgeCPE\left(e_{1}\right)+\ldots +edgeCPE\left(e_{g}\right).$$

To improve space efficiency, we modify the labelling scheme for the edge belonging to *case0*. If *e *∈ *NTE*(*v*) and *e *∈ *case*0, the destination node of *e *must be *v*. Thus, we can use a special character '?' to replace *PET*(*v*).

We model Pedigrees, Individuals, and Compact Path Encoding labels in our relational database with the following tables:

**Pedigree**(*PedigreeID*, *Name*)

**Individual**(*IndividualID*, *PedigreeID*, *MotherID*, *FatherID*, *Gender*, *Name*, *DOB*, *DOD*, etc.)

**CompactPathEncoding**(*PET*, *PedigreeID*, *NTE*, *IndividualID*)

### Generating CPE labels

**Figure 4:**
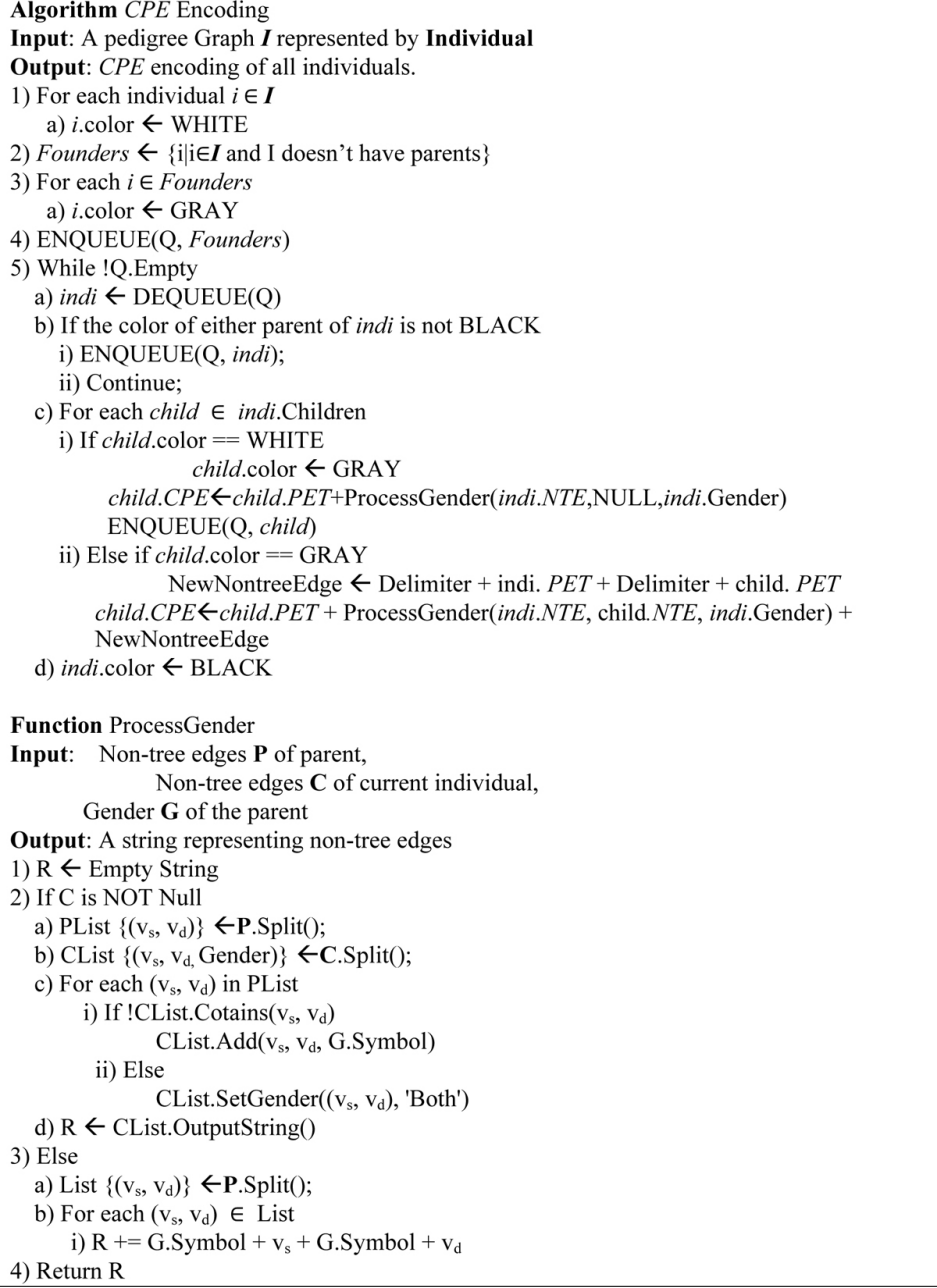
Algorithm: CPE Encoding.

The *CPE *encoding algorithm in Figure 4, which is a modification of Breadth First Search(BFS), starts from the founders of the pedigree graph and generates the *CPE *label for each node in the graph in breadth first order. During each round of the BFS, we make sure the parent node's *CPE *is finalized before finalizing the children node's *CPE*.

When the algorithm processes each child of the current individual, if a child has not been visited before, (i.e., the color of that child is white), the child inherits the non-tree edges of its parent and the color of the child is updated to gray. After that, the child is pushed to the end of the queue. On the other hand, if the color of a child is gray (i.e., this child has been visited before), the edge connecting the individual to this child must be a non-tree edge. Therefore, the non-tree edge set of this child is the union of the non-tree edge set of itself and the non-tree edge set of its parent, plus the new non-tree edge. After processing all the children of the current individual, we change the color of the individual to black, which means that this individual has been finalized. By enforcing the order of visiting individuals, we can avoid pushing the same individual into the queue for multiple times. Thus, the efficiency of encoding is improved.

Given a pedigree graph *G *= (*V*, *E*), *CPE *encoding and NodeCodes encoding have the same asymptotic time complexity, which can be expressed as *O*(|*E*| + |*V*|) since every vertex and every edge needs to be explored a constant number of times.

### Optimizing the size of CPE encoding

Given a pedigree graph, *G *= (*V*, *E*), the length of the PET codes of the nodes, and hence the size of the *CPE *encoding for G, differs by the choice of spanning tree that is used for PET codes.

**Figure 5:**
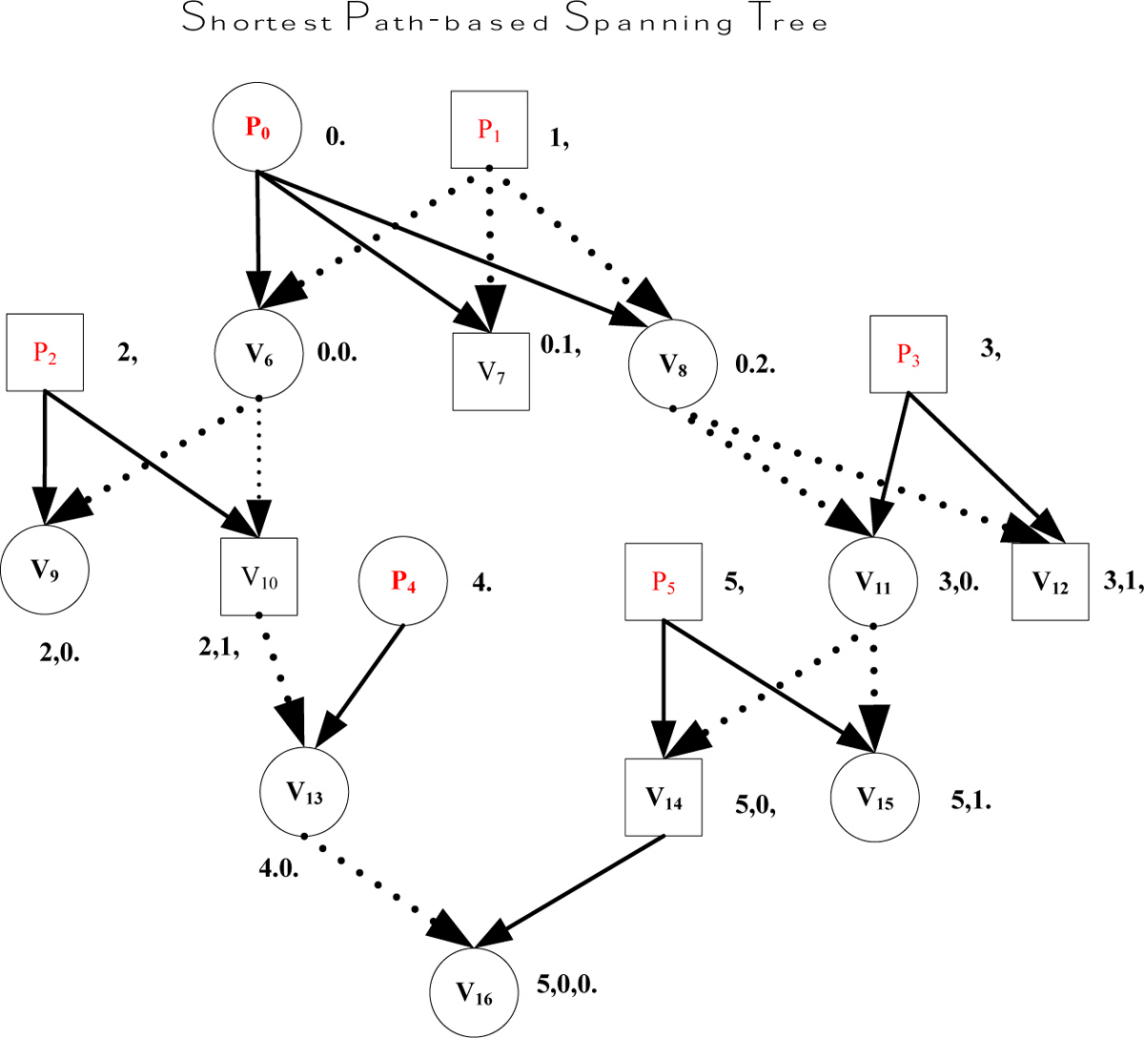
An example of shorter PET codes.

**Figure 6:**
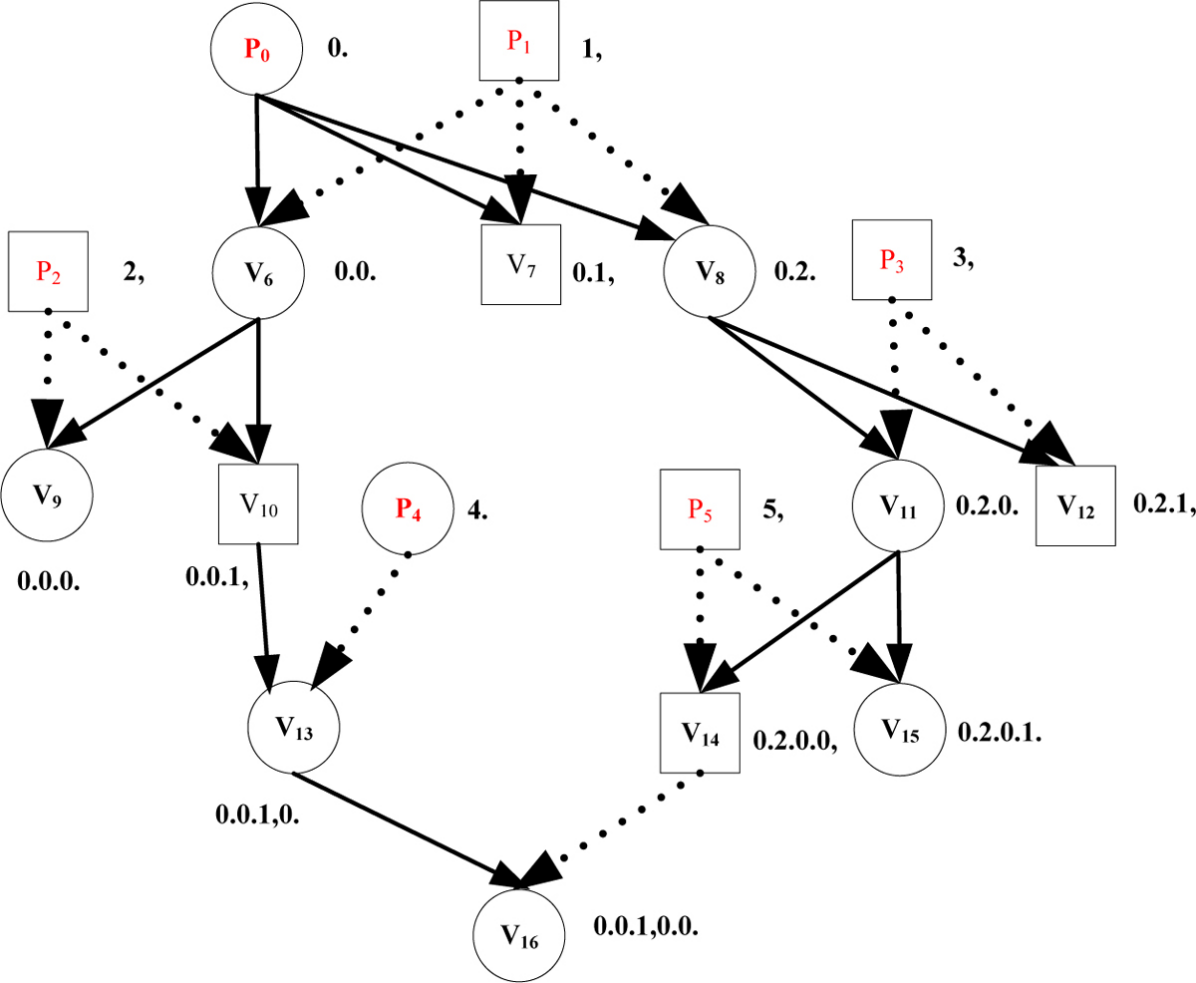
An example of longer PET codes.

**Example 3: **Consider the two different spanning trees shown in Figure 5 &6, for the pedigree graph in Figure 3. Note that in Figures 5 and 6, we omit the node for the unique (virtual) progenitor r, which is the root of the spanning tree, and the (tree) edges from r to the (actual) progenitors P_0_,..., P_5_.

We can see that for each node *v *in the pedigree graph, the size of the PET(*v*) in Figure 5, is less than or equal to the size of PET(*v*) in Figure 6. In fact, for the spanning tree shown in Figure 5, the sum of the lengths of tree edges from the root (or progenitors) to each other node (non-progenitors) is minimum of all alternative choices for tree edges for this pedigree graph. We refer to such spanning trees as *shortest path-based spanning tree*. As a matter of fact, the encoding algorithm in Figure 4 obtains a shortest path-based spanning tree, which also leads to the minimum total size for the *CPE *code as will be shown below.

For simplicity, we assume that each node has two incoming edges, except the founders who don't have parents. And we also ignore the gender delimiters. For an individual *v_i_*, we use |*PET*(*v_i_*)| to denote the length of *PET *code of v_i_, and *Des*(*v_i_*) to denote all descendants of *v_i_*. The number of descendants of *v_i _*is |*Des*(*v_i_*)|. A non-tree edge between individual *v_i _*and individual *v_j _*is denoted as *e_ij_*. *Len*(*e_ij_*) denotes the length of the encoding for the non-tree edge *e_ij_*, where *Len*(*e_ij_*) = |*PET*(*v_i_*)| + |*PET*(*v_j_*)|. Given a node *v_j _*which has an incoming non-tree edge *e_ij_*, the number of descendants of *v_j _*is |*Des*(*v_j_*)|. Thus, the contribution from e_ij _to *Space*(*G*), which is the size of the whole CPE code, can be computed as

$$S\left(e_{ij}\right)=Len{\left(e_{ij}\right)}^{*}\left(|Des\left(v_{j}\right)|+1\right)={\left(\left|PET\left(v_{i}\right)|+|PET\left(v_{j}\right)\right|\right)}^{*}\left(|Des\left(v_{j}\right)|+1\right)$$

Considering the PET codes for all nodes in *V *and the contribution from all non-tree(NTE) edges, we obtain the total length of CPE for *G*, *Space*(*G*), as follows:

(2) $$\begin{array}{ll} Space\left(G\right) & =\underset{1\leq k\leq |V|}{\sum }|PET\left(v_{k}\right)|+\underset{e_{ij}\in NE}{\sum }S\left(e_{ij}\right)\;\\ & =\underset{1\leq k\leq |V|}{\sum }|PET\left(v_{k}\right)|+\underset{e_{ij}\;\in NE}{\sum }Len{\left(e_{ij}\right)}^{*}\left(|Des\left(v_{j}\right)|+1\right)\;\\ & =\underset{1\leq k\leq |V|}{\sum }|PET\left(v_{k}\right)|+\underset{e_{ij}\in NE}{\sum }{\left(\left|PET\left(v_{i}\right)|+|PET\left(v_{j}\right)\right|\right)}^{*}\left(|Des\left(v_{j}\right)|+1\right)\;\\ & \; \end{array}$$

**Lemma 1: **For a pedigree graph G the spanning tree which minimizes the size of PET codes in G also minimizes the total size of CPE encoding for G.

**Proof**: First of all, an individual *v_i _*is chosen arbitrarily from the pedigree graph *G*. The individual *v_i _*can have at most two in-coming edges. In *G*, *Des*(*v_i_*) is fixed, because the ancestor-descendant relationship is defined by *G*. No matter which edge we choose as the non-tree edge, it is be inherited by all the descendants of *v_i_*. Thus, the (|*Des*(*v_i_*) + 1|) part in the formula 2 doesn't change.

Secondly, there are one in-coming tree edge and one in-coming non-tree edge for an individual *v_i_*. For the non-tree edge for *v_i_*, no matter which parent is chosen as the non-tree edge parent, the length of the non-tree edge doesn't change. Because if we choose mother as the non-tree parent, the PET of the individual will be equal to the PET of father concatenated by one encoding character specific for v_i_, then the length of the non-tree edge will be |*PET*(Mother)| + |*PET*(Father)| + 1. The result is the same if we choose father as the non-tree parent, and it is always equal to |*PET*(Mother)| + |*PET*(Father)| + 1.

**Proposition 1: **For a pedigree graph *G*, using the breadth first search (BFS) to encode the PET code minimizes *Space*(*G*), which is the size of CPE encoding for G.

**Proof: **The spanning tree built by BFS has the shortest path from root to each node by the definition of BFS. Since the length of the PET code for a node is determined by the length of the path from the root to the node, the *PET *code of each node is also minimal for the tree produced by BFS. Since the algorithm in Figure 4 uses BFS, and using Lemma 1, the algorithm also minimizes the total size of CPE encoding for G.

## Path construction

The paths between an individual *n *and one of its ancestors *p *can be divided into two sets: tree paths and non-tree paths. A tree path only contains tree edges, while a non-tree path has at least one non-tree edge.

**Lemma 2**: If *PET*(*p*) is a prefix of *PET*(*n*), there is a tree-path from *p *to *n*.

**Proof**: According to the *PET *encoding scheme for a tree *T*, if *PET*(*p*) is a prefix of *PET*(*n*), it means that *p *is an ancestor of *n *along the tree. Therefore, we can conclude that there must be a path on the tree from *p *to *n*.

**Figure 7:**
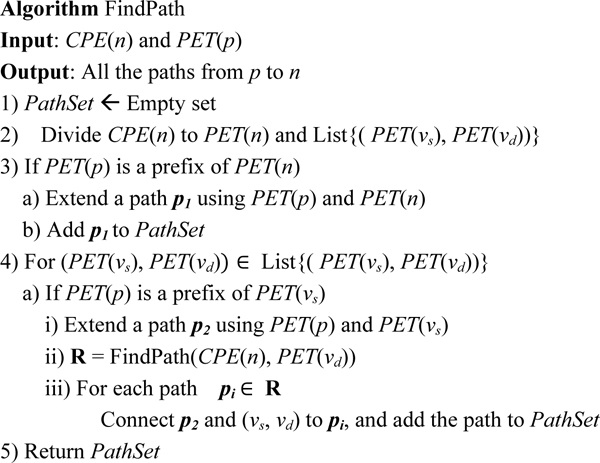
Algorithm: FindPath.

Now, we leverage the property of the *PET *code to reconstruct the tree path from one ancestor node to a given node. To obtain all non-tree paths, we design a recursive procedure to find all the paths containing at least one non-tree edge. The basic idea is to start with a node *u *in the path which contains a non-tree edge, and this node has an outgoing non-tree edge ending at *v*. Then we decompose the finding path procedure into two parts, the path from the common ancestor to the node *u *and the path from *v *to the individual. In this way, we use the *CPE *code to recursively reconstruct all the non-tree paths. Figure 7 is the pseudo-code algorithm.

**Example 4: **Let's take the node *P_6 _*in Figure 3 as an example and show how to use FindPath algorithm in Figure 7 to find all paths from *P_1 _*to *P_6_*.

$$\begin{array}{c} PET\left(P_{1}\right):1,\\ CPE\left(P_{6}\right):1,1.\$0.0,0.\$1,1.\#1,\;\#0.0,\;\#1,0.\;\#0.0,0.\\ PET\left(P_{6}\right):1,1\\ \left(PET\left(v_{s}\right)\to PET\left(v_{d}\right)\right):\left\{0.0,0.\to 1,1.\right\},\;\left\{1,\to 0.0,\right\},\;\left\{1,0.\to 0.0,0.\right\} \end{array}$$

According to step 3, *PET*(*P_1_*) is a prefix of *PET*(*P_6_*), thus there is a tree path from *P_1 _*to *P_6_*. Then, we process all non-tree edges. For the edge represented by {0.0,0.→1,1.}, *PET*(*P_1_*) is not a prefix of the string '0.0,0.', so we skip this edge. For the edge represented by {1,→0.0,}, *PET*(*P_1_*) is a prefix of '1,' (actually, *PET*(*P_1_*) is '1,'), but the tree path *p_2 _*for this edge is NULL. Recursively we can find the paths from '0.0,' (corresponding to node *P_2_*) to *P_6_*.

$$\begin{array}{c} PET\left(P_{2}\right):0.0,\\ CPE\left(P_{6}\right):1,1.\$0.0,0.\$1,1.\#1,\#0.0,\#1,0.\#0.0,0.\\ PET\left(P_{6}\right):1,1.\\ \left(PET\left(v_{s}\right)\to PET\left(v_{d}\right)\right):\left\{0.0,0.\to 1,1.\right\},\left\{1,\to 0.0,\right\},\left\{1,0.\to 0.0,0.\right\} \end{array}$$

In terms of finding the paths from node *P_2 _*to *P_6_*, *PET*(*P_2_*) is not a prefix of *PET*(*P_6_*), so there is not a tree-path. But, for the non-tree edge represented by {0.0,0.→1,1.}, *PET*(*P_2_*) is a prefix of '0.0,0.', so there is a tree path from *P_2 _*to '0.0,0.', following by a non-tree edge from '0.0,0.' to *P_6_*. Thus, we obtain a non-tree path from *P_2 _*to *P_6 _*(represented as 0.0,→0.0,0.→1,1.). Once the recursion is done, we have a non-tree path from *P_1 _*to *P_6 _*(represented as 1,→0.0,→0.0,0.→1 ,1.) which is obtained in according to the non-tree edge{1,→0.0,}.

Similarly, we process the non-tree edge {1,0.→0.0,0.}, and obtain a non-tree path from *P_1 _*to *P_6 _*(represented as 1,→1,0. →0.0,0.→1,1.). Totally, we have obtained three paths from *P_1 _*to *P_6_*.

$$\begin{array}{c} p_{1}:1,\to 1,1.\\ p_{2}:1,\to 0.0,\to 0.0,0.\to 1,1.\\ p_{3}:1,\to 1,0.\to 0.0,0.\to 1,1. \end{array}$$

Similarly, we obtain two paths from *P_1 _*to *P_5_*.

$$\begin{array}{c} q_{1}:1,\to 0.0,\to 0.0,0.\to 0.0,0.0,\\ q_{2}:1,\to 1,0.\to 0.0,0.\to 0.0,0.0, \end{array}$$

## Inbreeding coefficients calculation

After obtaining the *CPE *label for each node in pedigree graphs, we use Wright's formula [6] presented in section 2 to calculate inbreeding coefficients. For the inbreeding coefficient of an individual, Wright's Formula requires identifying: parents of the individual, common ancestors of parents, and non-overlapping pairs of paths from these common ancestors to both parents.

**Definition (Non-overlapping pair of paths)**: Let {*p_1_*, *p_2_*} be a pair of paths from a common ancestor *c *to mother *m *and father *f *of an individual. The pair {*p_1_*, *p_2_*} is *non-overlapping *if *c *is the only node in common to *p_1 _*and *p_2_*. Otherwise, the pair of paths {*p_1_*, *p_2_*} is overlapping, and the nodes in common to paths *p_1 _*and *p_2 _*other than *c *are called the *crossover *nodes, which are also common ancestors for *m *and *f*.

### Identifying father and mother

Given *CPE*(*n*), which is the CPE code of an non-root individual *n*, we first identify the *PET *of the father and mother from *CPE*(*n*). Since one node can only have at most two incoming edges in a pedigree graph, we have the following lemma.

**Lemma 3**: If an individual only has one parent, then there must be a tree edge connecting the individual and its parent. Otherwise, (individual has two parents) the edges connecting the individual to its parents must be one tree edge and one non-tree edge.

**Figure 8:**
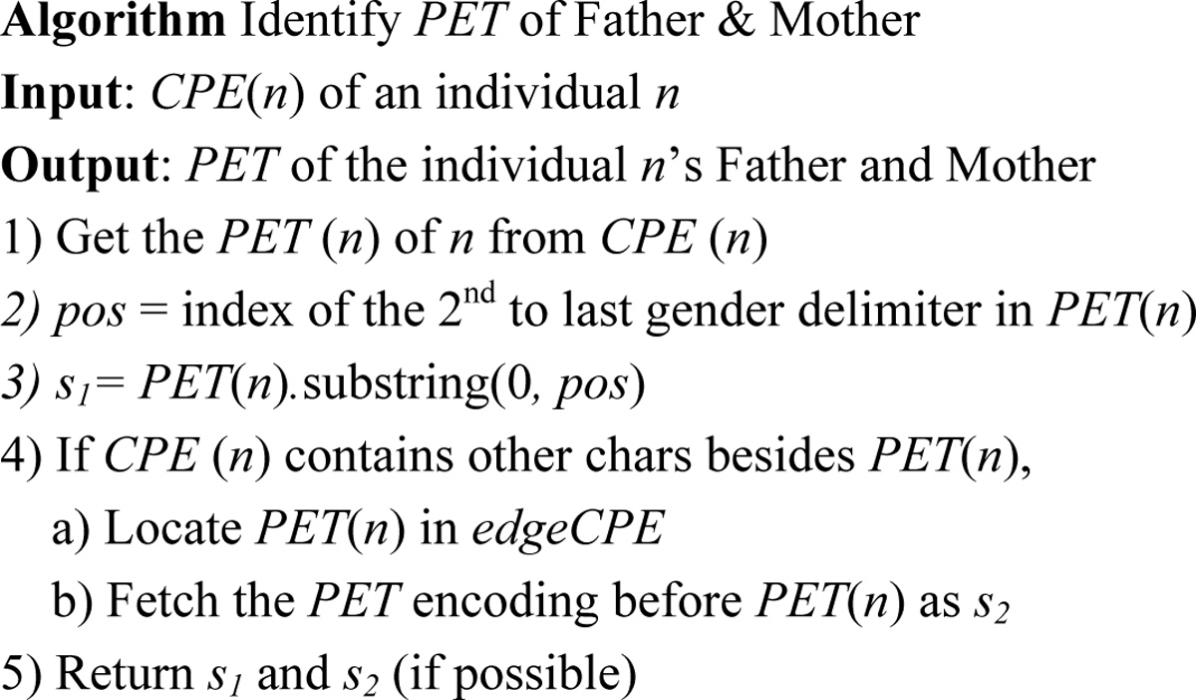
Algorithm: Identify PET of Father and Mother.

Lemma 3 is utilized in the algorithm shown in Figure 8 which outlines identifying the *PET *for mother and father of an individual efficiently.

**Example 5: **Let's take the node *P_7 _*in Figure 3 as an example and illustrate how to use the algorithm to find the parents of *P_7_*.

$$CPE\left(P_{7}\right):0.0,0.0,0,\$1,1.\$0.0,0.0,0,\#0.0,0.\#1,1.\text{\&}1,\text{\&}0.0,\text{\&}1,0.\&0.0,0.$$

In the first step, we get the *PET*(*P_7_*), which is '0.0,0.0,0,'. And we obtain the position of the 2^nd ^to last gender delimiter in *PET*(*P_7_*), and we get the substring of *PET*(*P_7_*) which ends with the 2^nd ^to last gender delimiter in *PET*(*P_7_*). As a result, we have '0.0,0.0,' which is the *PET *label for node *P_5_*.

In the second step, we find that *CPE*(*P_7_*) still contains other symbols, then we try to locate *PET*(*P_7_*) in this string. According to the lemma above, we can conclude that there is only one appearance of this *PET*(*P_7_*) in this string. Next, we fetch the *PET *label just before *PET*(*P_7_*), which is '1,1.' and this is the *PET *label for node *P_6_*. Thus, both the *PET *codes for the mother and father of node *P_7 _*are obtained.

**Figure 9:**
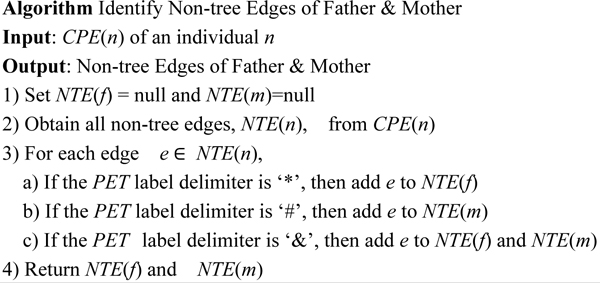
Algorithm: Identify Non-tree Edges of Father and Mother.

After obtaining the father and mother's *PET*, we need to obtain the non-tree edges for the father and mother respectively from *CPE*(*n*). According to the different types of *PET *label delimiters, (i.e. {'$', '*', '#', '&'}), we can efficiently identify the non-tree edges inherited from the father or mother. Figure 9 shows the algorithm.

**Example 6: **For node *P_7_*, all non-tree edges are represented as

$$e_{0}=\$1,1.\$0.0,0.0,0,\;\;e_{1}=\#0.0,0.\#1,1.\;\;e_{2}=\&1,\&0.0,\;\;e_{3}=\&1,0.\&0.0,0.$$

We obtain *NTE*(*P_5_*) = {*e*_2_, *e*_3_} and *NTE*(*P_6_*) = {*e*_1_, *e*_2_, *e*_3_}.

Then, we use a generic *PET *label delimiter '%' to construct *CPE*(*f*) and *CPE*(*m*). The resulting *CPE *labels are capable for identifying common ancestor and corresponding paths from each common ancestor to *f *and *m*. Thus, we can get the *CPE *code of *P_5 _*and *P_6_*.

$$\begin{array}{c} CPE\left(P_{5}\right):0.0,0.0,\%1,\%0.0,\%1,0.\%0.0,0.\\ CPE\left(P_{6}\right):1,1.\%0.0,0.\%1,1.\%1,\%0.0,\%1,0.\%0.0,0. \end{array}$$

### Identifying common ancestors

For an individual *n *having parents *f *and *m*, we can use *CPE*(*f*) and *CPE*(*m*) to obtain all the common ancestors of *f *and *m*, as shown in Lemma 4 below. Let Prefix(s) be the set of all prefixes of string s. For clarity, a prefix of a string s = *s_1_...s_k _*is a string s' = *s_1_...s_j_*, where *j *≤ *k*.

**Lemma 4**: Given an individual *n*, *CPE*(*n*) contains all the *PET *of *n*'s ancestors.

**Proof**: If a node *u *is an ancestor of *n*, then the paths from *u *to *n *can be divided into two sets: tree path and non-tree path(s). A tree path only contains tree edges, while a non-tree path has at least one non-tree edge.

Given the node *n *which has the non-tree edges {*e_1_*, *e_2 _*... *e_k_*} in *PS*(*n*), the *CPE *encoding for *n *is represented as *CPE(n) *= *PET(n) *+ *edgeCPE(e_1_) *+ ... + *edgeCPE(e_k_) *where *edgeCPE*(*e_i_*) = {*PET*(*v_si_*), *PET*(*v_di_*)} for *e_i _*= (*v_si_*, *v_di_*), 1 ≤ i ≤ k

If there is a tree path from *u *to *n*, then *PET*(*u*) is a prefix of *PET*(*n*), denoted as *PET*(*u*) ∈ Prefix(*PET*(*n*)).

If there is no tree path from *u *to *n*, then there exists a vertex *x *in the list of non-tree edges listed in *CPE*(*n*) such that *u *is connected to vertex *x *by a tree path. *PET*(*u*) must be in the set A(*n*) = {Prefix(*PET*(*x*)) | (*x*,*v*) is a non-tree edge in the list of non-tree edges for *n*}. Hence, if *u *is an ancestor of *n*, then *PET*(*u*) is either in Prefix(PET(*n*)) or it is in A(*n*). Thus, the *CPE(n) *contains all the PET codes of *n*'s ancestors.

**Example 7: **To obtain all the common ancestors of *P_5 _*and *P_6_*, we first calculate the ancestors of *P_5 _*and *P_6 _*respectively. If the node *u *has both tree path and non-tree paths to *n*, then the node *u *can be obtained from *PET*(*n*), and we don't need to keep *PET*(*u*) in the prefix sets obtained from the non-tree edges of *n* (see Table 1).

**Table 1:** 

Ancestor(*P5*)	Prefix(*PET*(*P5*))	0.	0.0,	0.0,0.	0.0,0.0,
	
	A(*P5*)	1,	1,0.		
		
Ancestor(*P6*)	Prefix(*PET*(*P6*))	1,	1,1.		
	
	A(*P*6)	0.	0.0,	0.0,0.	1.0.

The common ancestors of *P_5 _*and *P_6 _*are obtained from the intersection of these two sets as shown in Table 2.

**Table 2:** 

Individual *n*	*P_1_*	*P_0_*	*P_2_*	*P_3_*	*P_4_*
*PET*(*n*)	1,	0.	0.0,	1,0.	0.0,0.

As we can see from Figure 3, the above result shows all the common ancestors of *P_5 _*and *P_6_*.

### Finding path-pairs

**Figure 10:**
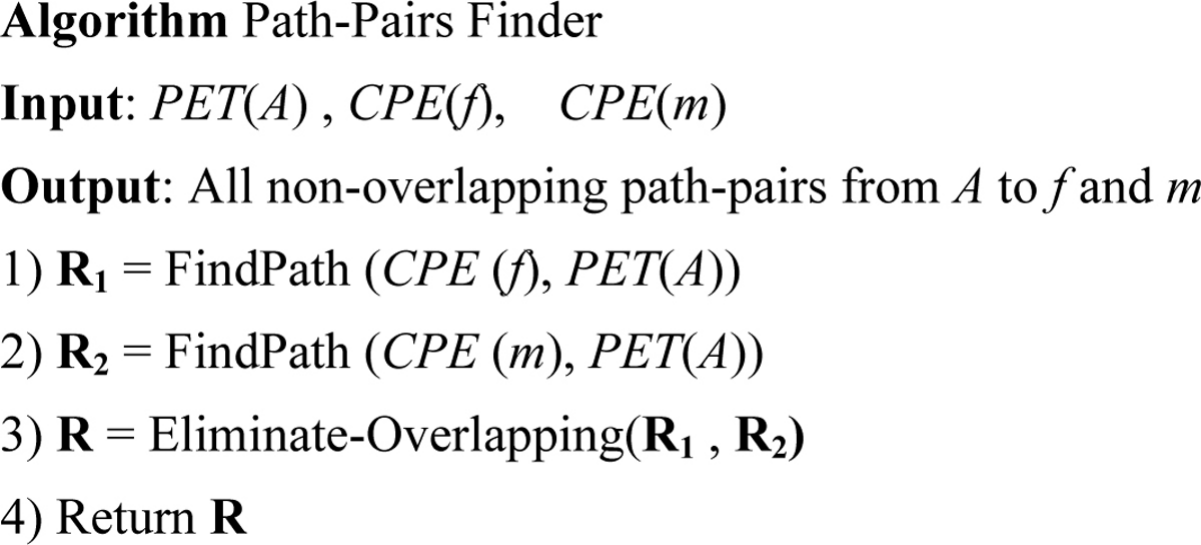
Algorithm: Path-Pairs Finder.

After getting all the common ancestors of the father *f *and mother *m *of an individual, we re-construct all the non-overlapping pairs of paths which are from a specific common ancestor *A *to the father *f *and mother *m *based on *PET*(*A*), *CPE*(*f*), and *CPE*(*m*). The outline of Path-Pairs Finder is shown in Figure 10.

### Finding paths for multiple ancestors

When the father *f *and mother *m *have more than one ancestor, we use memorization technique to solve the path finding problem regarding all ancestors. More specifically once we have finished finding all the paths from one common ancestor to *f *(or *m*), we will store all these paths for future usage. The next time we meet the same ancestor, we can get all the paths without calculating them again.

### Identifying non-overlapping path-pairs

After we get the two sets of the paths, we can use all common ancestor of the father *f *and mother *m *to build an inverted index for these paths. The keys of the inverted index are the *PETs *of the common ancestors, and the elements are the paths ID containing the corresponding common ancestor.

**Example 8: **For the paths {*p_1_*, *p_2_*, *p_3_*, *q_1_*, *q_2_*} in example 4, we can build the following index using all the common ancestors for *P_5 _*to *P_6_*. The common ancestors are listed in Table 3.

**Table 3:** 

Individual *n*	*P_1_*	*P_0_*	*P_2_*	*P_3_*	*P_4_*
*PET*(*n*)	1,	0.	0.0,	1,0.	0.0,0.

The inverted index is as follows (see Table 4).

**Table 4:** 

1,	*p_1_*, *p_2_*, *p_3_*, *q_1_*, *q_2_*
0.0,	*p_2_*, *q_1_*
1,0.	*p_3_*, *q_2_*
0.0,0.	*p_2_*, *p_3_*, *q_1_*, *q_2_*

**Figure 11:**
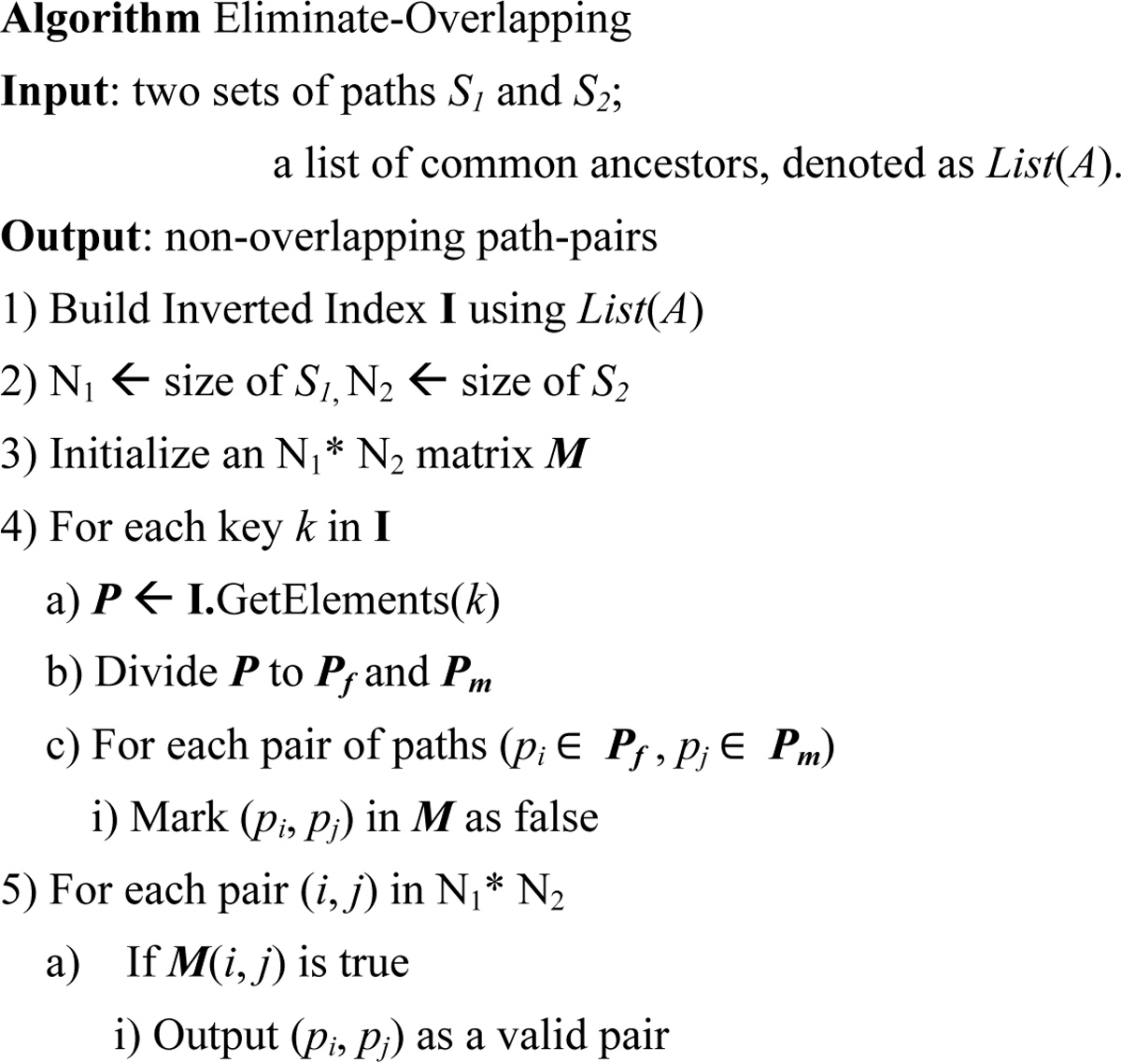
Algorithm: Eliminate-Overlapping.

Using the above inverted index, and we propose the algorithm in Figure 11 to eliminate the overlapping paths. After applying the algorithm in Figure 11 to eliminate the overlapping paths, we can get the following table for the matrix *M* (see Table 5).

**Table 5:** 

	*p_1_*	*p_2_*	*p_3_*
*q_1_*	True	False	False
*q_2_*	True	False	False

Thus, the non-overlapping pairs of paths are {*p_1_*, *q_1_*} and {*p_1_*, *q_2_*}. After obtaining all non-overlapping path-pairs for the ancestor *P_1_*, we follow Step 2.c) and 2.d) in the Inbreeding Coefficient algorithm in Figure 1 to obtain the node *P_1_*'s contribution to the node *P_7_*'s inbreeding coefficient.

### Improved CPE

One of the bottlenecks in inbreeding computations with CPE is to identify the pairs of paths between the mother and father of an individual and their common ancestor that contribute to the inbreeding. From the algorithm in Figure 10, we can see that only the non-overlapping pairs of paths contribute to the final inbreeding coefficient. If a common ancestor cannot provide any non-overlapping pair of paths, then we call this common ancestor a **nonessential ancestor**. We can identify such ancestors beforehand, and filter them out before the costly path-pairs checks for improving the efficiency of the inbreeding coefficient computation with CPE.

For clarity, let *Sub_G*(*v, m, f*) denote the unique *minimal *sub-digraph of *G *that contains all the paths in *G *from the node *v *to nodes *m *and *f*.

**Lemma 5: **In *Sub_G*(*u, m, f*), if node *u *only has one child *v *(where both *u *and *v *are common ancestors of *m *and *f*), then *u *must be a nonessential common ancestor.

**Proof**: Given two nodes *u *and *v *which are common ancestors of *m *and *f*, if node *u *is the parent of *v*, we know *N_m_*(*u*) ≥ *N_m_*(*v*), because $N_{m}\left(u\right)=\sum_{v\in children\;of\;u}N_{m}\left(v\right)$. The result also holds for *N_f_*(*u*) and *N_f_*(*v*). Since node *v *is the only child of *u *in the context of *Sub_G*(*u, m, f*), it is true that *N_m_*(*u*) = *N_m_*(*v*) and *N_f_*(*u*) = *N_f_*(*v*). If *N_m_*(*u*) = *N_m_*(*v*), it means that every path from *u *to *m *passes through *v*. If *N_f_*(*u*) = *N_f_*(*v*), we can conclude that every path from *u *to *f *must pass through *v*. Therefore, all pairs of paths from ancestor *u *to *m *and *f *are overlapping, because they all pass the ancestor *v*. Therefore, *u *must be a nonessential ancestor for *m *and *f *in terms of inbreeding calculation.

Based on Lemma 5, before checking the overlapping relationship between every pair of paths, we can check the number of children of the current ancestor *u*. If there exists only one child of the current ancestor *u*, the ancestor *u *is nonessential and we can skip it.

**Example 9: **In Figure 3, both *P_2 _*and *P_4 _*are common ancestors of *P_5 _*and *P_6_*. While *P_2 _*has only one child *P_4 _*in *sub*_*G*(*P_2_*, *P_5_*, *P_6_*). According to Lemma 5, we can eliminate *P_2 _*from the common ancestors set safely.

One problem to be addressed is how to identify the children of one individual. When calculating the inbreeding coefficient of one individual, the mother and father for that individual needs to be obtained first. In the meanwhile, an adjacency list can be maintained to record the parent-children relationship. If the individuals are processed in topological order, we can make sure that we get all the parent-children relationship for all the ancestors of current individual. Therefore, when processing the current individual, we can take advantage of this parent-children relationship and prune the nonessential ancestors.

### Complexity analysis

#### Time complexity

Let *k *be the average number of *PET*s in the *CPE *of one individual whose inbreeding coefficient is being calculated, and *s *is the length of the longest *PET *among these *PET*s. Then, the identifying parents step takes O(*k*) time. The common ancestors of parents are obtained by getting the intersection of the unique prefix sets of mother and father. To get the unique prefix set of one parent, we process the *PET*s one by one. Thus, it may take as long as O(s*k*log(s*k)) for one parent. We can maintain the prefix set as sorted, in such case the intersection of these two sets only takes O(s*k) operations. Let *n *be the number of paths from the root to that individual. Identification of the non-overlapping pairs of paths could take as much as O(*n*^2^) time. Therefore, the total time complexity for calculating the inbreeding coefficient for one individual is O(*k*) + O(s*k*log(s*k)) + O(*n*^2^) = O(s*k*log(s*k)+*n*^2^).

For calculating the inbreeding coefficients for multiple individuals, we can use the property that siblings share exactly the same inbreeding coefficient to prune repeated calculation. Suppose we want to calculate the average inbreeding coefficient for a set of *t *individuals, and the average number of children of each pair of parents is *m*. Then the total time complexity could be O((s*k*log(s*k)+*n*^2^)*t/*m*).

#### Space complexity

In this subsection, we first analyse the space complexity theoretically and then provide an example to illustrate that the worst case we have is tight. Suppose there are V individuals and E edges in the pedigree graph. The upper bound of the length of one CPE code is equal to the maximum length of PET code times the maximum number of PET codes in one CPE code. The maximum length of the PET code could be (V - 1), and the maximum number of PET code in one CPE code could be 1 + 2*(E - V), since there could be at most (E - V) non-tree edges in one CPE code and each of the edges is consisted of two PET codes. The space complexity is equal to (V - 1) * (1 + 2*(E - V)) = O(V^2^(E - V)). Since in a pedigree graph the total number of edges is less than or equal to 2*V, the total space complexity should be O(V^3^).

**Figure 12:**
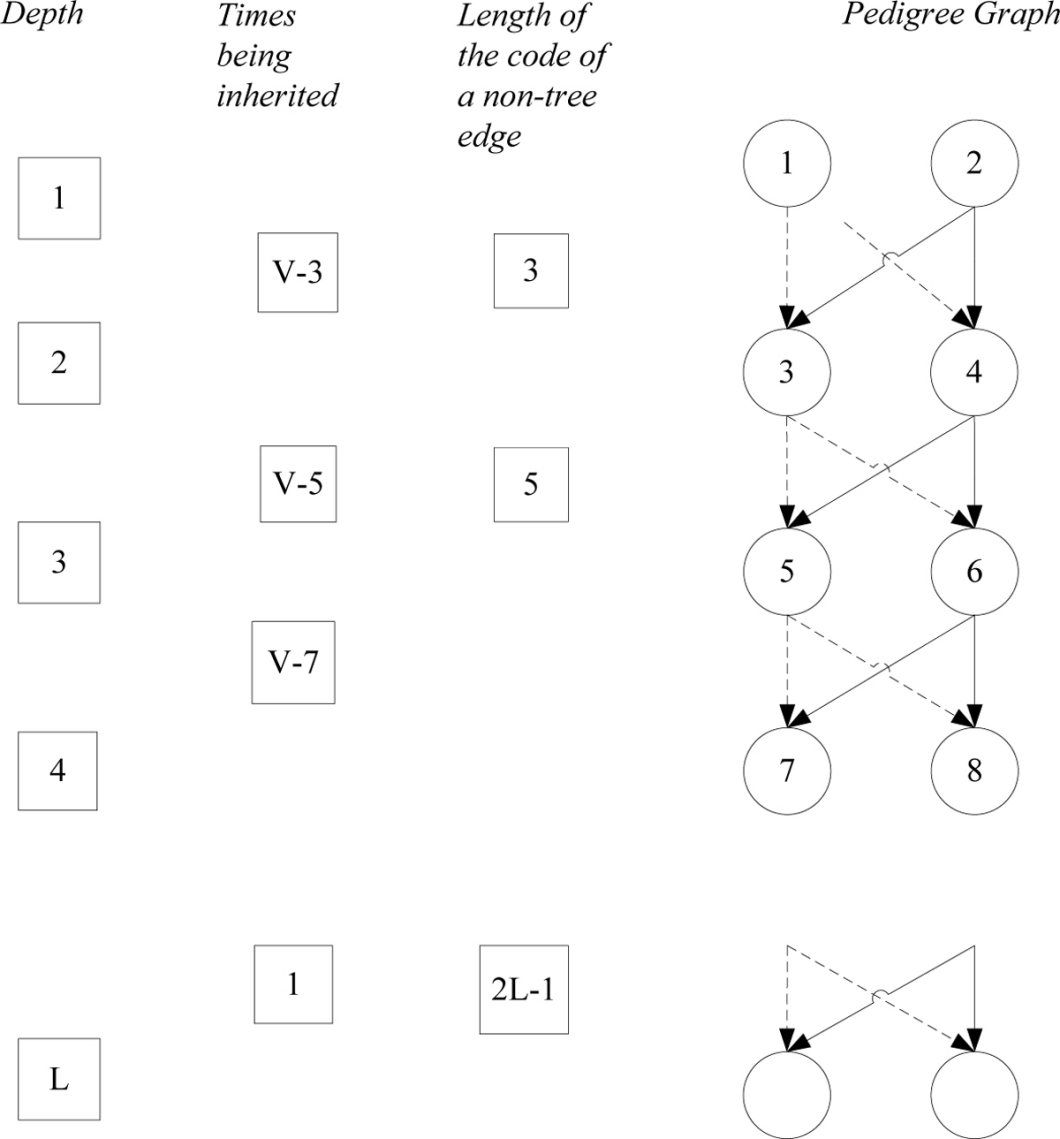
An example of pedigree illustrating a worst-case scenario.

Although the bound we got from the above analysis looks relatively loose, from the following example we can see that it's tight in the worst case. In Figure 12, an extreme case of the pedigree graph has been shown. The first column shows the length of the PET code at each level. The second column shows how many times that the non-tree edges are inherited at that level. For example, the non-tree edge between individual 1 and individual 3 will be inherited by all the individuals except individual 1, 2 and 4. Since there are V individuals totally, that edge will be inherited by V - 3 times. The third column shows the length of non-tree edge at that level. For example, the length of the PET code for individual 1 is 1 and the length of the PET code for individual 4 is 2, so the non-tree edge between individual 1 and individual 4 is equal to 1 + 2 = 3.

Therefore, the total length of the encoding for non-tree edges is equal to Σ (the length of each non-tree edge * the number of times that edge is repeated). The result is 3*(V - 3) + 5*(V - 5) + ... + (2L - 1)*1, where L is equal to V/2. After we calculate the result, it's O(V^3^). Since the space complexity for only the non-tree edges part is already O(V^3^), the space complexity for the whole CPE encoding certainly should be O(V^3^).

## Experiments

In this section we show the effectiveness of our CPE for inbreeding query evaluation by comparing with the NodeCodes-based computation [18,19] and traditional iterative method used in existing systems [20,21].

### Experimental data

The Cleveland Clinic's (CCF) Familial Polyposis Registry [5], which we used as our real data set, is the largest inherited colorectal cancer registry in the United State and the second largest in the world. The Polyposis Registry captures complex pedigree and clinical data such as demographic characteristics, pedigree relations, distribution of polyps, cancer sites, surgical procedures and medical treatments. Our real dataset consists of 654 pedigrees containing 8345 individuals. The largest one consisted of 118 individuals spanning 8 generations. The 2^nd ^largest one consisted of 115 individuals spanning 7 generations. There are 32 pedigrees having average inbreeding coefficients larger than 0. The size of these pedigrees ranges from 7 to 118. The average inbreeding coefficient of these 32 pedigrees ranges from 0.0015 to 0.0417.

In order to test the scalability of our approach for calculating inbreeding coefficients on large pedigrees, we used a population simulator implemented in [18] to generate arbitrarily large pedigrees. The population simulator is based on the algorithm for generating populations with overlapping generations in Chapter 4 of [22] along with the parameters given in appendix B of [23] to model the relatively isolated Finnish Kainuu subpopulation and its growth during the years 1500-2000. An overview of the generation algorithm was presented in [18]. The parameters include: starting/ending year, initial population size, initial age distribution, marriage probability, maximum age at pregnancy, expected number of children by time period, immigration rate, and probability of death by time period and age group.

For our synthetic data, large pedigrees were generated by running the simulator from the year 1500 with an initial population 30, and immigration rates from 0.005 to 0.01, while the maximum number of individuals to generate was raised from 1000 to 10,000. For each parameter setting (pedigree size and immigration rate), we chose 3 different random seeds to generate three pedigrees, and obtain the average number of founders and average inbreeding coefficient for each setting. In this paper, we use average inbreeding coefficient as one of the characteristics for pedigree data. The detailed result is listed in Table 6.

**Table 6:** Average inbreeding coefficient for synthetic pedigrees having different immigration rate and size

Pedigree size	Immigration rate	# of founders	Average inbreeding coefficient
1000	0.005	196	0.0032986
	0.01	270	0.0022222

2500	0.005	404	0.0035005
	0.01	606	0.0015536

5000	0.003	554	0.0044207
	0.005	736	0.0030604
	0.007	921	0.0021039
	0.008	994	0.0014392
	0.01	1145	0.0009371

10000	0.005	1409	0.00022236
	0.01	2226	0.0006543

The average inbreeding coefficient is 0.03 for the Dunker population in Pennsylvania and 0.04 for islanders on Tristan da Cunha [24]. The average inbreeding coefficient is 0.00210 for Norwegian 19th century data [25]. The average inbreeding coefficient for 435777 Utah Mormons in [26] is 0.000106.

### Experimental setup

We tested the effectiveness of our method using C# .NET 4.0 and SQLServer 2008. We implemented *CPE*, improved version of *CPE *and NodeCode labelling algorithms and used strings to store *CPE *and NodeCodes with the sibling numbers encoded in a base-64 representation. All queries were run on cold cache and the test machine was a 2.93GHZ Intel(R) Xeon with 48GB RAM running Windows Server 2008 R2.

We compared the execution time required to calculate inbreeding coefficients by the recursive method, the path-counting method using NodeCodes, and the path-counting method using *CPE and improved-CPE*. We analysed the effects of pedigree size (# of individuals) and the average inbreeding coefficient value. We refer to the recursive method as *Iterative*, the path-counting method using NodeCodes as *NodeCodes*, the path-counting method using *CPE *as *CPE*, and the path-counting method using *CPE *and eliminating nonessential ancestor as *I-CPE *respectively.

### Experiments on real data

The Cleveland Clinic's (CCF) Familial Polyposis Registry [5] is used as our real data set. Among 654 real pedigrees, only two pedigrees have more than 115 individuals and no pedigrees in the 76-114 range. Therefore, the two largest real pedigrees are used for average space efficiency comparison. For one individual, the average length of *CPE *is 15.67 bytes, while the average length of NodeCodes is 15.84 bytes. Since the real pedigrees are very small, *CPE *achieves small improvement over NodeCodes. In the next section, we use synthetic data to demonstrate that the space improvement from *CPE *grows as the pedigree size grows. Then, the two largest real pedigrees are used for average computation efficiency performance comparison, and the results are shown in Table 7. As can be seen, *CPE *method performs best for the two largest real pedigrees among three methods.

**Table 7:** Time cost results on real data

	*CPE*	*NodeCodes*	*Iterative*
Average	0.61	1.35	1.33

### Experiments on synthetic data

In this section, we demonstrate the scalability and efficiency of *CPE *with respect to space cost and computation cost on synthetic data.

**Figure 13:**
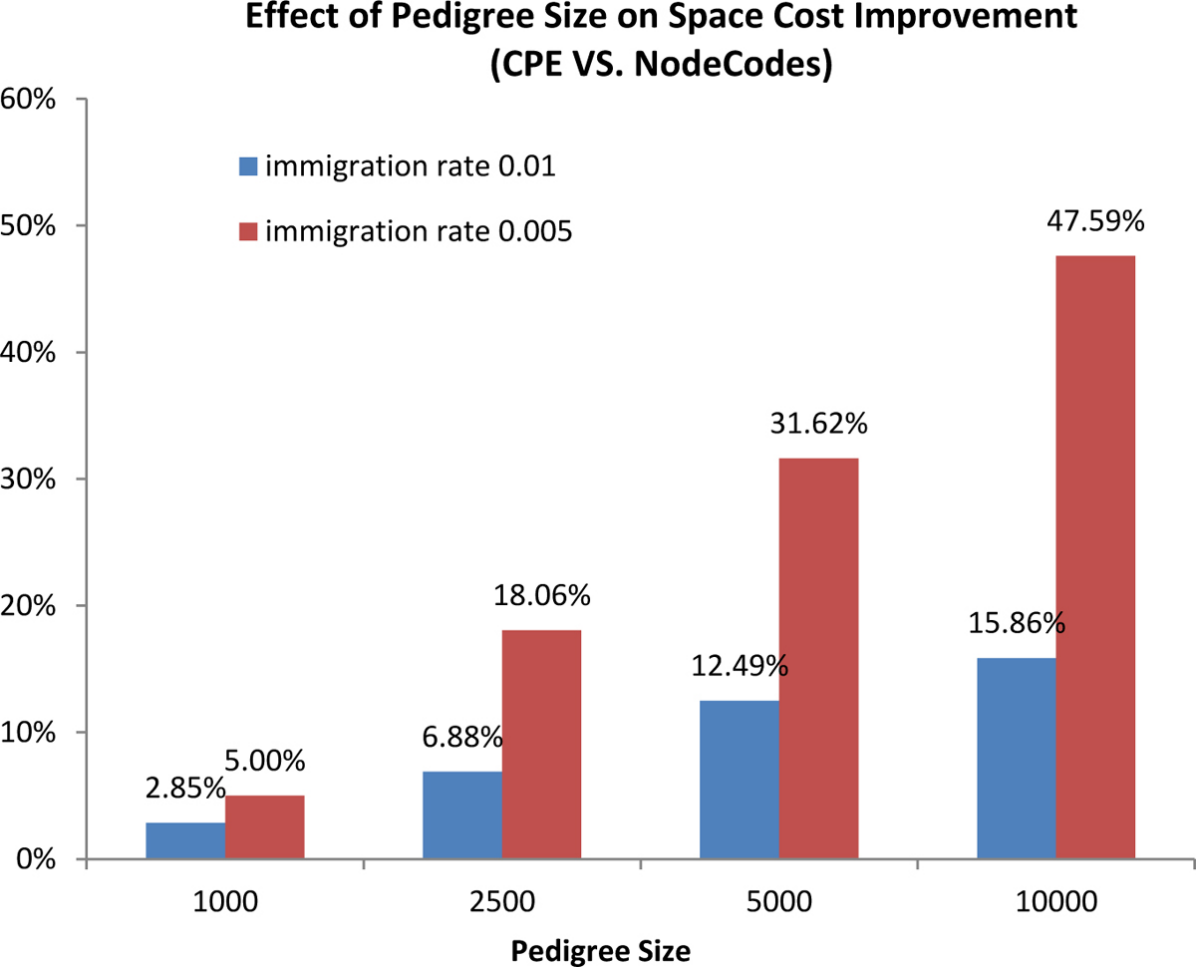
Effect of pedigree size on the space cost improvement of CPE over NodeCodes.

In the first experiment, we used two different immigration rates (0.005, 0.01) to generate pedigrees for each of the different pedigree sizes. In terms of space efficiency, we compared the total length of *CPE *with NodeCodes. Figure 13 shows the effect of pedigree size on the space cost improvement of *CPE *over NodeCodes. The improvement of *CPE *grew increasingly larger as the pedigree size increased, from a comparable amount 5.00% on the smallest pedigree to 47.59% on the largest pedigree when the immigration rate is 0.005.

**Figure 14:**
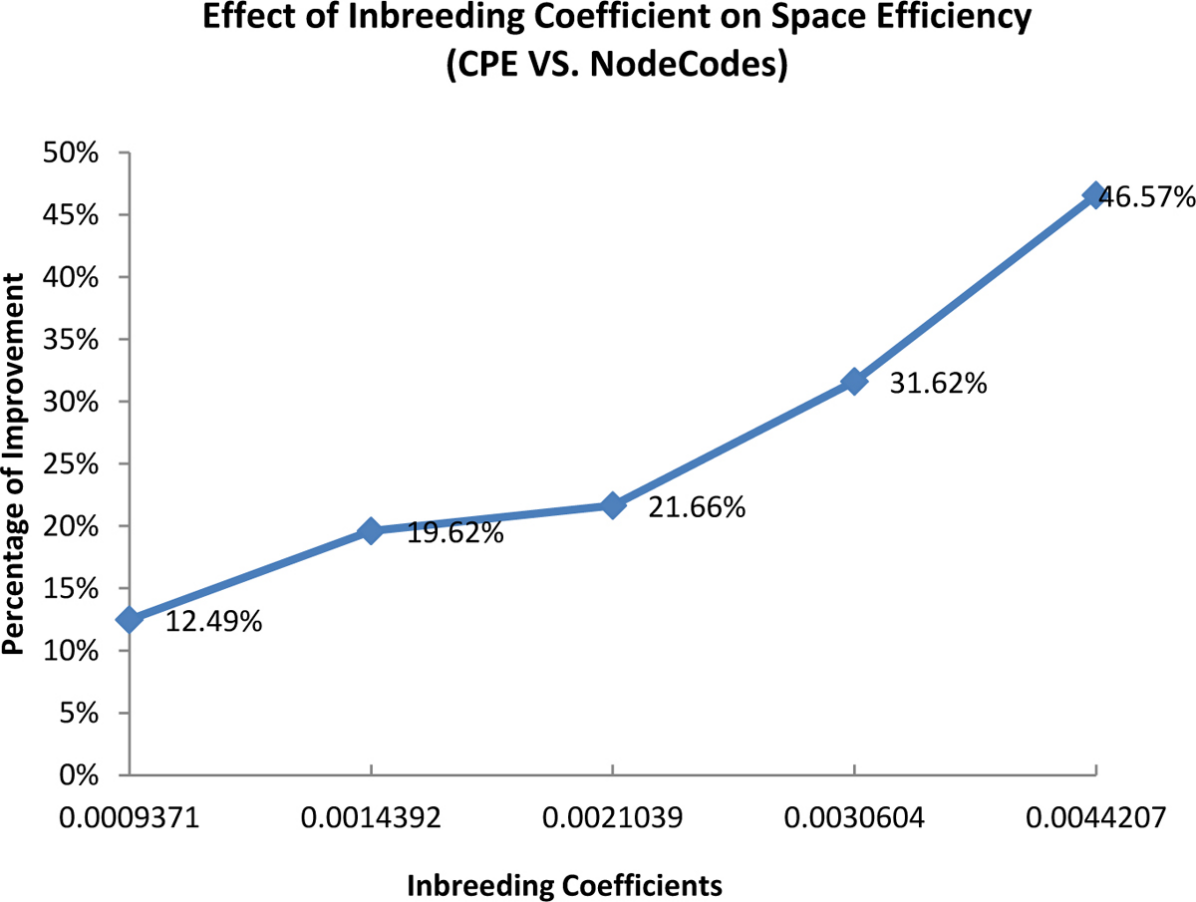
Effect of inbreeding coefficient on the space cost improvement of CPE over NodeCodes.

In the 2nd experiment, 5 pedigrees (size 5000) having different inbreeding coefficients which were generated by setting different immigration rates (0.003, 0.005, 0.007, 0.008, 0.01) were selected to compare the total length of *CPE *with NodeCodes. Figure 14 shows the effect of inbreeding coefficients on the space cost improvement of *CPE *over NodeCodes. The improvement of *CPE *grew increasingly larger as the inbreeding coefficients increased, from a comparable amount 12.49% on the pedigree having average inbreeding 0.00079371 to 46.57% on the pedigree having average inbreeding 0.0044207.

**Figure 15:**
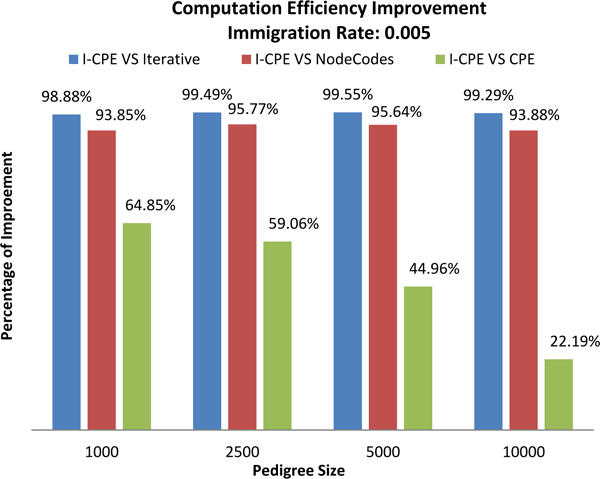
Effect of pedigree size on performance improvement for immigration rate 0.005.

**Figure 16:**
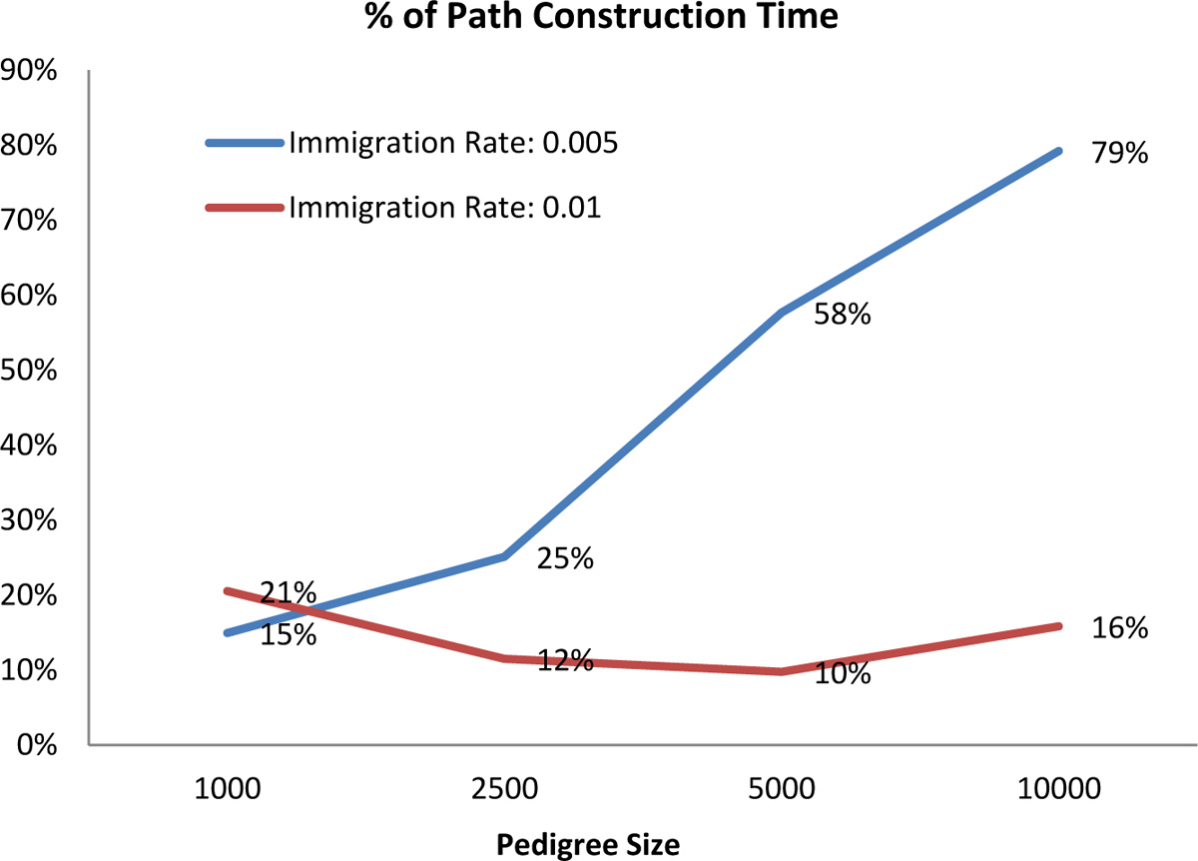
Percentage of path construction time of the total computation time by I-CPE method.

In order to see how the performance improvement of *CPE *scales with the pedigree size, we used two different immigration rates (0.005, 0.01) to generate pedigrees for each of the different pedigree sizes. The results shown in Figures 15 and 16 are the average performance for running the experiment three times with three different pedigrees for each parameter.

As shown in Figure 15, for the pedigrees generated by setting immigration rate 0.005, the improvement of *I-CPE *over *Iterative *is over 98%; the improvement of *I-CPE *over *NodeCodes *is over 93%; and the improvement of *I-CPE *over *CPE *ranges from 22.19% to 64.85%.

In Figure 15, as we notice that the advantage of *I-CPE *over the original *CPE *is decreasing as the size of the pedigree graph increases. Our *CPE *method for computing the inbreeding coefficient consists of two time-consuming parts, which are the path construction part and non-overlapping paths checking part. The improved version of *CPE *method only tries to optimize the non-overlapping paths checking part. The problem here is that the path construction time dominates the total computation time when the size of the pedigree graph becomes large, which can be seen from Figure 16. In another word, the non-overlapping paths checking part becomes less significant. Thus, the optimization on that part becomes less significant. In fact, when the size of the pedigree becomes large, the total number of paths is exponentially increasing, and that's reason that the path construction time dominates the total computation time finally.

**Figure 17:**
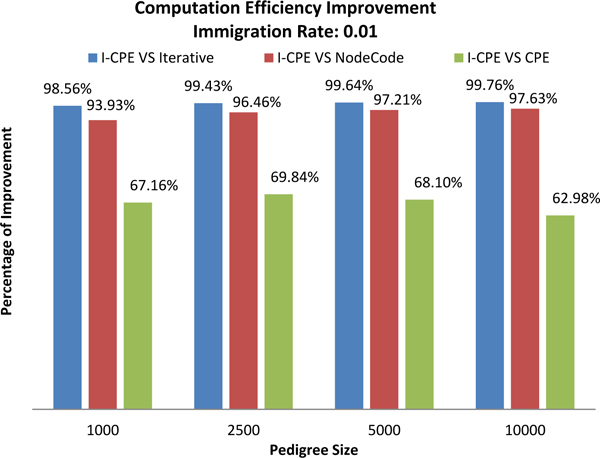
Effect of pedigree size on performance improvement for immigration rate 0.01.

Figure 17 shows the percentage improvement of *I-CPE *over *Iterative, NodeCodes, and CPE *with respect to increasing pedigree size generated by setting immigration rate 0.01. Performance gains of *I-CPE *over *CPE *ranges from 67.16% to 62.98%.

In the last experiment, 5 pedigrees (size 5000) having different inbreeding coefficients were selected for demonstrating the effect of inbreeding coefficients on the computation improvement of *CPE *over NodeCodes. Figure 17 shows the effect of inbreeding coefficients on the time cost improvement of *I-CPE *over *CPE *and NodeCodes.

**Figure 18:**
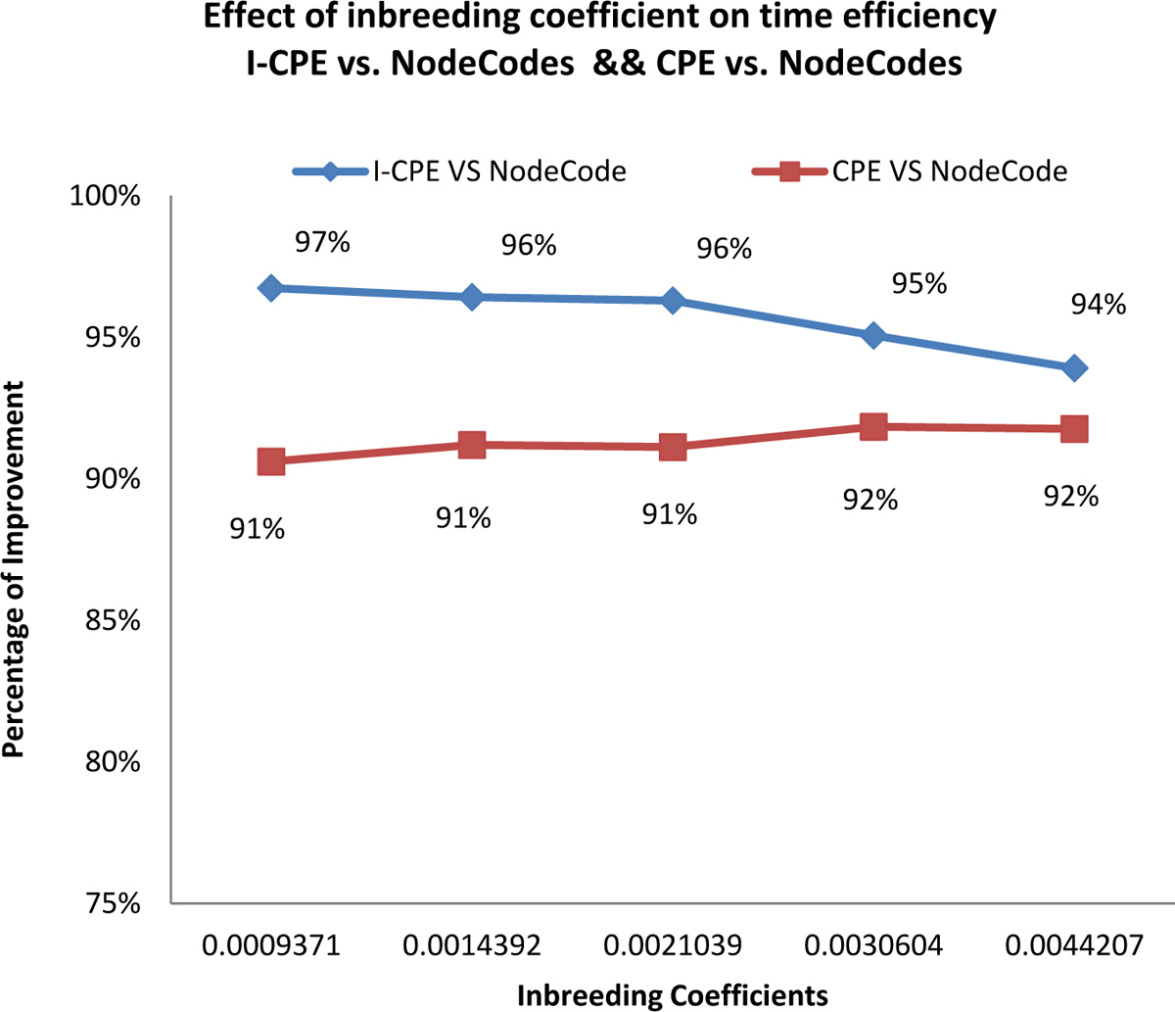
Effect of inbreeding coefficient on the performance improvement of I-CPE and CPE over NodeCodes.

As shown in Figure 18, the computation efficiency improvement of *CPE *over *NodeCodes *grows increasingly larger as the inbreeding coefficients increase, and it is over 90% even for the smallest inbreeding coefficient tested. On the other hand, the computation efficiency improvement of *I-CPE *over *NodeCodes *decreases as the inbreeding coefficients increase. The reason is that as the inbreeding coefficient increases, the complexity of the pedigree graph increases, which means the total number of paths in the pedigree graph grows exponentially. As we have analyzed previously, the path construction time dominate the total computation time eventually, so the relative performance of *I-CPE *over *NodeCodes *decreases. In the extreme case, when the inbreeding coefficient becomes even large, the performance of *I-CPE *and *CPE *tends to be same.

## Further improvements

To reduce the space cost of labels for a graph, one possible solution is to compact the graph itself with smaller number of nodes edges. For pedigree data, instead of using nodes to represent individuals, we can compact families, i.e., parents and their children, as nodes in the pedigree graph. The directed edges in this graph between the nodes representing families represent relationships between families. That is, there is an edge between two nodes if there is a shared individual between the two families (e.g. a child in one family may be the parent in another family). Such a representation of pedigrees, using family nodes is used in [19], and shows a significant improvement over NodeCodes in terms of space and time requirements for path-based computations over large pedigrees. Utilizing *CPE *together with Family-level representation of pedigree graphs for the computation of inbreeding coefficients, and other path-based computations on large pedigrees will make the scalability and the performance efficiency of *CPE *based computation even more pronounced.

## Other related work

There are also a few other commercial and academic software packages for calculating inbreeding coefficients, including FSpeed [20], LaoTzu's Animal Register [21], Cyrillic [27]. One of the most popular commercial packages is Cyrillic 2.1, which can calculate inbreeding and crossovers from phenotype data, but provides no support for structure-based querying and only supports pedigrees of up to 10,000 individuals. Cyrillic 3 supports larger pedigrees, but simply uses MENDEL [28] for pedigree analysis such as inbreeding, which uses a technique that is known to only work for calculating inbreeding of small to medium-sized pedigrees [29]. Another popular commercial pedigree software product is Progeny [30]. As compared to NodeCodes [18], *CPE *is a more compact encoding, and instead of keeping one code for each path to a node, it constructs the paths as needed from one compact code of the node. As compared to Extended Greedy for DAGs (EGDL) [17] the encoding for *CPE *is more compact, and it is tailored for applications for large pedigrees. Using *PET *delimiters for *CPE*, the non-tree edges inherited via paternal and maternal paths to an individual can be distinguished efficiently.

## Conclusions

We have proposed a new compact path encoding (*CPE*) scheme for pedigree graphs for efficient evaluation of path based computations on pedigree data. The compact path encoding is also applicable to other DAGs in general. We used computation of inbreeding coefficients of an individual using Wright's path counting formula to demonstrate the effectiveness and the efficiency of *CPE*. We have presented algorithms to generate all paths to an individual from its ancestors from the *CPE *code of the node corresponding to the individual, as well as algorithms to identify common ancestors, overlapping paths, etc. We also implemented and tested our method using both real and synthetic data of various sizes to test scalability. Experimental results show that the use of *CPE *for inbreeding coefficients calculation performs significantly better than Nodecodes and iterative method both in terms of time and space requirements. Our future work includes (i) further improvements on compact encodings, and (ii) developing scalable methods for calculating inbreeding coefficients and other genetic computational problems including generalized kinship and identity coefficients, using compact encodings and path-counting formulas.

## Competing interests

The authors declare that they have no competing interests.

## Authors' contributions

This paper is co-authored by Lei Yang, En Cheng and Meral Ozsoyoglu. Developing algorithms (i) for encoding graphs using CPE, (ii) for constructing paths from CPE encoding, (iii) for the computations of inbreeding coefficient efficiently, (iv) the improved CPE algorithms, and (v) space and time complexity results are all done in collaboration among the three authors led by Meral Ozsoyoglu. Experiments are also designed by all three authors, En Cheng generated the synthetic data used by the experiments, and Lei Yang coded and ran the experiments. Manuscript has been read and approved by all authors and that all authors agree to the submission of the manuscript to BMC Bioinformatics journal.
